# Machine learning approaches for predicting arsenic adsorption from water using porous metal–organic frameworks

**DOI:** 10.1038/s41598-022-20762-y

**Published:** 2022-09-30

**Authors:** Jafar Abdi, Golshan Mazloom

**Affiliations:** 1 Faculty of Chemical and Materials Engineering, Shahrood University of Technology, Shahrood, Iran; 2 Department of Chemical Engineering, Faculty of Engineering, University of Mazandaran, Babolsar, Iran

**Keywords:** Environmental sciences, Engineering, Mathematics and computing, Nanoscience and technology

## Abstract

Arsenic in drinking water is a serious threat for human health due to its toxic nature and therefore, its eliminating is highly necessary. In this study, the ability of different novel and robust machine learning (ML) approaches, including Light Gradient Boosting Machine (LightGBM), Extreme Gradient Boosting, Gradient Boosting Decision Tree, and Random Forest was implemented to predict the adsorptive removal of arsenate [As(V)] from wastewater over 13 different metal–organic frameworks (MOFs). A large experimental dataset was collected under various conditions. The adsorbent dosage, contact time, initial arsenic concentration, adsorbent surface area, temperature, solution pH, and the presence of anions were considered as input variables, and adsorptive removal of As(V) was selected as the output of the models. The developed models were evaluated using various statistical criteria. The obtained results indicated that the LightGBM model provided the most accurate and reliable response to predict As(V) adsorption by MOFs and possesses R^2^, RMSE, STD, and AAPRE (%) of 0.9958, 2.0688, 0.0628, and 2.88, respectively. The expected trends of As(V) removal with increasing initial concentration, solution pH, temperature, and coexistence of anions were predicted reasonably by the LightGBM model. Sensitivity analysis revealed that the adsorption process adversely relates to the initial As(V) concentration and directly depends on the MOFs surface area and dosage. This study proves that ML approaches are capable to manage complicated problems with large datasets and can be affordable alternatives for expensive and time-consuming experimental wastewater treatment processes.

## Introduction

As a highly toxic material, arsenic is distributed all over environmental waters. Arsenic can be produced naturally through biological activity and earth crust. Also, it can be caused by human activity such as agriculture, mineral extraction, and discharge of industrial wastewater^1^. Inorganic arsenic mainly exists in two forms; arsenite [As(III)] and arsenate [As(V)]. Arsenate is the main species found in natural surface water bodies, while arsenite predominantly exists in the groundwater^2^. Both of them are highly toxic, but As(III) is approximately 60 times more toxic than As(V)^3^. Generally, As(III) holds neutral and un-dissociated forms, therefore its removal is very challenging^4^ and it is required first to oxidize arsenite to arsenate for its effective removal. Arsenic is a global threat to human health. Long-term exposure to arsenic, mainly through contaminated water and food, can cause severe diseases such as kidney, liver, skin, and lung cancers^5^. World Health Organization (WHO) has set the maximum level of 10 µg/L for arsenic in drinking water^6^. Therefore, effective removal of this heavy metal is still a vital task. Different technologies have been developed for arsenic removal, including ion exchange, biological techniques, coagulation, precipitation, reverse osmosis, filtration, and adsorption^7,8^. Among these technologies, adsorption over the porous adsorbents is generally one of the most promising methods due to the high efficiency, cost affordable, and mild operating conditions^9^. Different porous adsorbents have been developed and studied for arsenic removal, such as zeolites, natural clay, carbonous materials, metal oxides, and metal–organic frameworks (MOFs)^10,11^. Recently, MOFs, as a new class of porous materials, have been attracted much interest in different fields^12–18^. MOFs consist of metal ions or clusters, which are coordinated through organic linkers. Owning to their characteristic features; i.e., large surface area, high porosity, adjustable pore size, high crystallinity, good thermal and chemical stability^19,20^. So far, many studies have shown the remarkable ability of different MOFs to adsorb various contaminants, such as heavy metals^5,21,22^. For example, the performance of ZIF-8 was evaluated in the adsorptive removal of As(III) and As(V) by Jian et al.^23^. The authors have reported the maximum adsorption capacity of 49.49 and 60.03 mg/g for As(III) and As(V), respectively, at pH = 7 and room temperature. UiO-66 was successfully synthesized by Audu et al.^24^ for adsorption of As(V) and As(III). They reported that faster and more efficient adsorption obtained as the pore sizes increased by reducing particle sizes. Li et al.^25^ was compared the performance of acetate modulated MOF-76(Y) with pristine MOF-76(Y) in the adsorptive removal of As(V) in the alkaline solutions. It was shown that the acetate modulated sample exhibited a higher pore volume and smaller particle size, which indicated excellent performance in the adsorption of arsenate with a maximum adsorption capacity of 201.46 mg/g. The superior performance of pristine MIL-88A(Fe) and MIL-88A(Fe) decorated on cotton fiber was reported by Pang et al.^26^ in the adsorption of As(III) and As(V).

Employing MOFs in the practical application requires many challenges and obstacles to be overcome. Evaluation of the MOFs’ efficiency involves conducting the experiments, which are the most expensive and time-consuming steps. In addition, the results obtained for the removal of the contaminants in laboratory operations cannot be scaled up to real plants. While studying the effects of different operating variables is necessary for control and consequently optimization in large-scale processes. The development of mathematical models is the first attempt to study the various processes widely studied by different researchers^27–31^. However, providing mathematical models for complex processes is extensively CPU- and time-consuming requiring a lot of time and effort. Fortunately, other approaches based on machine learning (ML) have been developed for modeling and simulation of such complex processes. These robust alternative approaches can predict complicated processes without solving theoretical equations. Nowadays, ML methods have been utilized in various areas due to their excellent performance with acceptable accuracy and reliability^32–38^.

In this study, we employ new approaches based on the ML for predicting As(V) adsorption from wastewater using MOFs at different operating conditions. The main novelty of the current work is the implementation of new innovative models that are efficient for managing extensive data collections. So far, the models based on the ML methods have not been previously employed for estimating As(V) removal over MOFs. Thus, a large dataset assigned to the As(V) adsorption by different MOFs adsorbents were collected at various conditions, including adsorbent dosage, arsenic concentration, contact time, temperature, solution pH, adsorbent surface area, and the presence of anions. Then, four powerful models Light Gradient Boosting Machine (LightGBM), Extreme Gradient Boosting (XGBoost), Gradient Boosting Decision Tree (GBDT), and Random Forest (RF) were implemented for predicting As(V) adsorption efficiency.

## Theory of the utilized model

### Light gradient boosting machine (LightGBM)

The LightGBM is developed according to the most basic concepts of gradient learning^39^. Comparing the LightGBM and the XGBoost throws light on the LightGBM’s better efficiency and consumption of less memory. These advantages expedite the training phase of the model development^40^. Dividing eigenvalues, the LightGBM form ‘k’ different bins. Doing so, a histogram having a total width of ‘k’ can be constructed. As soon as the procedure mentioned above is completed, there would be no need for any ensemble with pre-sorted results. The resulting values could be stored in eight-bit memory space as an integer value. Therefore, the amount of memory needed for keeping the calculated values would be dipped drastically, and consequently, such an approach reduces the preciseness of the resulting model. The LightGBM also benefits from the Leaf-wise problem-solving method. It has been seen in investigations that the leaf-wise strategy is much more robust and expeditious than any other traditional strategy. The most salient and affecting factor in making the leaf-wise strategy more powerful and reliable than any level-wise approach is the fact that all the leaves of a specific layer will possibly be taken into consideration for calculations, which decreases memory allocation^41^.

### Extreme gradient boosting model (XGBoost)

To find the minimum answer for a series of objective functions defined for an ensemble, classification and regression trees (CARTs) can be used expeditiously. Among all various forms of gradient boosting structures, the XGBoost method could be mentioned as a highly efficient tree-shaped approach. Typically, a CART model comprises three primary layers. Firstly, the main nodes could also be referred to as the root layer. Secondly, the interiors or internal nodes and what is located at the third layer could be named as the leaves or leaf nodes. A range of processes known as binary decision-making operations is responsible for dividing the root node and forming the internal nodes. These processes expeditiously develop the internal nodes from data sets made available in the root node. Finally, the classification operations will be completed in leaves of the modeling tree, resulting in the final classes. The robustness and the accuracy of every model could be improved by introducing various ensembles to the CARTs and developing them by assigning specified weight factors. The mentioned weight factor will determine how much an ensemble could affect the final result of the model^42^.

### Gradient boosting decision tree (GBDT)

In contrast with the Adaboost approach, the Gradient Boosting model utilizes the previously made residual errors of its precursor learners^43^. As in this method, a loss function is minimized during the model development procedure; it can be contemplated as a kind of decent gradient approach^44^. The currently presented study seeks to benefit from a combination of the Gradient Boosting approach and the decision trees, known as the gradient boosting decision tree (GBDT) method. Suppose an ensemble of experimentally obtained data with the form of $\left\{ {\left( {x_{1} ,y_{1} } \right), \left( {x_{2} ,y_{2} } \right),\left( {x_{3} ,y_{3} } \right), \ldots ,\left( {x_{n} ,y_{n} } \right)} \right\}$, the GBDT’s steps could be presented as follows^41^:

Step A. Initialization of $f_{0} \left( x \right)$.

Step B. Iteration on tree learners from b = 1 to b = B.

B1. Calculation of negative gradient (Z_l_).

B2. Setting $G_{b} \left( x \right)$ (regression tree) to the targets (Z_l_, l = 1,…,N).

B3. Determination of the size of each decent gradient.

B4. Continuously update $$f_{b} \left( x \right)$$.

Step C. the output corresponding to each data point of (x, b) will be $f_{B} \left( x \right)$.

For developing a predictive model, a range of hyper-parameters had to be assigned, such as the number of decision tree learners, a subset of the ensemble for initial feeding to the learners, the upper limit of the allowable depth, the lowest number of leaves, number of features, and number of data points in the separated sample as the sample split^45^.

### Random forest (RF)

The random forest modeling approach is fabricated from a combination of various decision trees. The random forest will train each tree simultaneously with other trees. However, it does not mean that trees have equal importance. In this predicting method, the algorithm is responsible for determining the superiority of every individual tree^46^. Additionally, to manage different features, the RF is enabled to select various features by implementing a built-in property of the RF classifier. This property will help the RF model to determine features without the elimination of some parameters and lowering the dimension of a complex problem^47^. Furthermore, a process known as bagging, which is the abbreviated version of bootstrap aggregating, is employed by the RF model to prevent similarity between trees in the forest and preserve their diversity. In the model development, the tree’s population is typically determined for the model as an inputted integer. Afterward, data points will be discretized into various subgroups according to the number of required trees. As a method for randomized sampling, bagging will try to use approximately 30 percent of data points in the training phase of every individual subtree. The remaining 70 percent of data points must be considered out-of-bag (OOB) data points.

## Models development

### Data assembling

**Figure 1:**
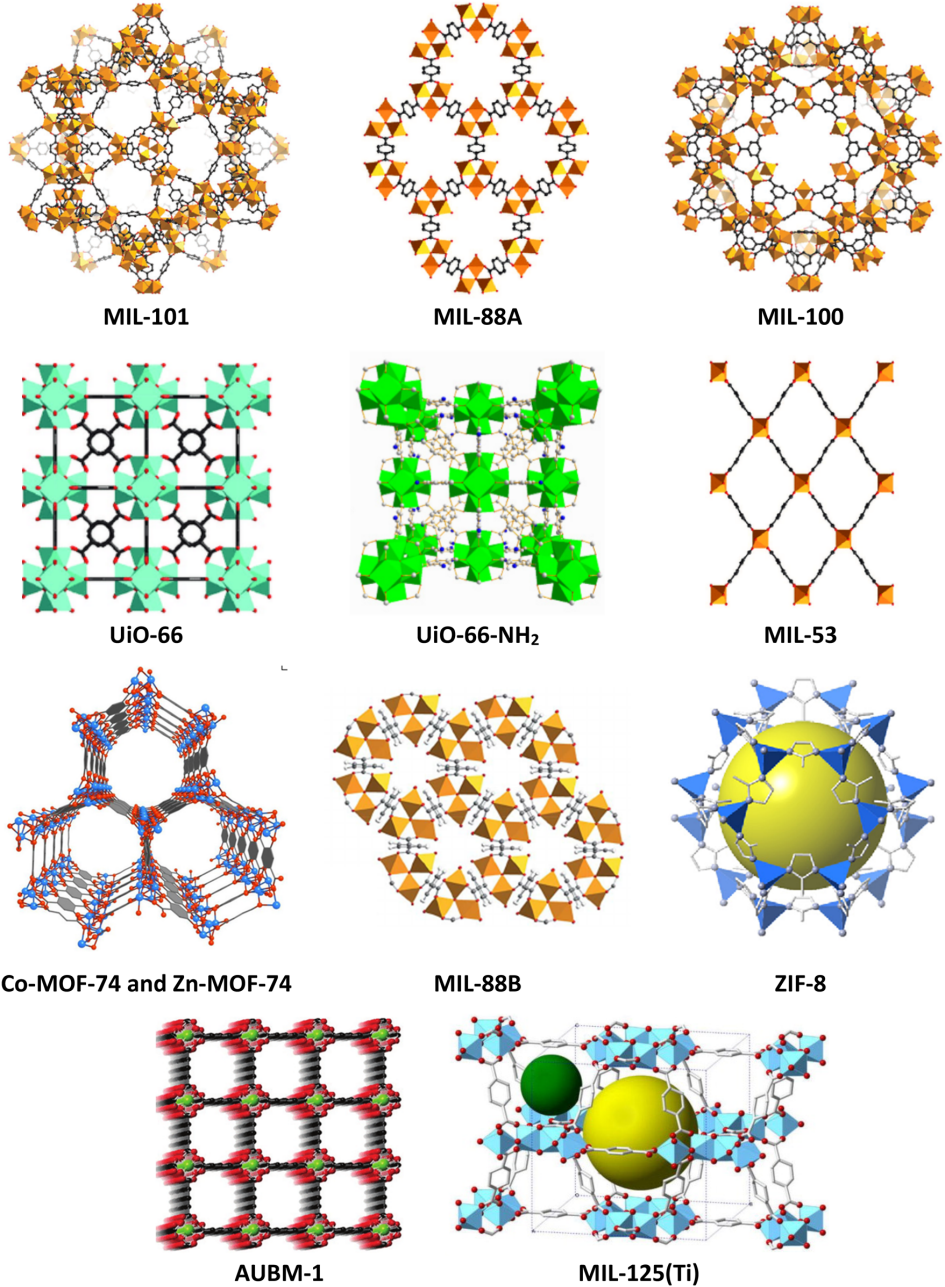
The framework structure of the investigated MOFs.

A large dataset consisting of 280 experimental dataset of As(V) elimination by various MOFs was collected using well-documented literature. The investigated MOFs include MIL-101(Fe), MIL-88A, MIL-100(Fe), UiO-66, UiO-66-NH_2_, MIL-53(Fe), Co-MOF-74, Zn-MOF-74, MIL-88B, ZIF-8, AUBM-1, GUT-3, and MIL-125(Ti)^4,23,48–57^. The structures of the selected MOFs are schematically presented in Fig. 1.

**Figure 2:**
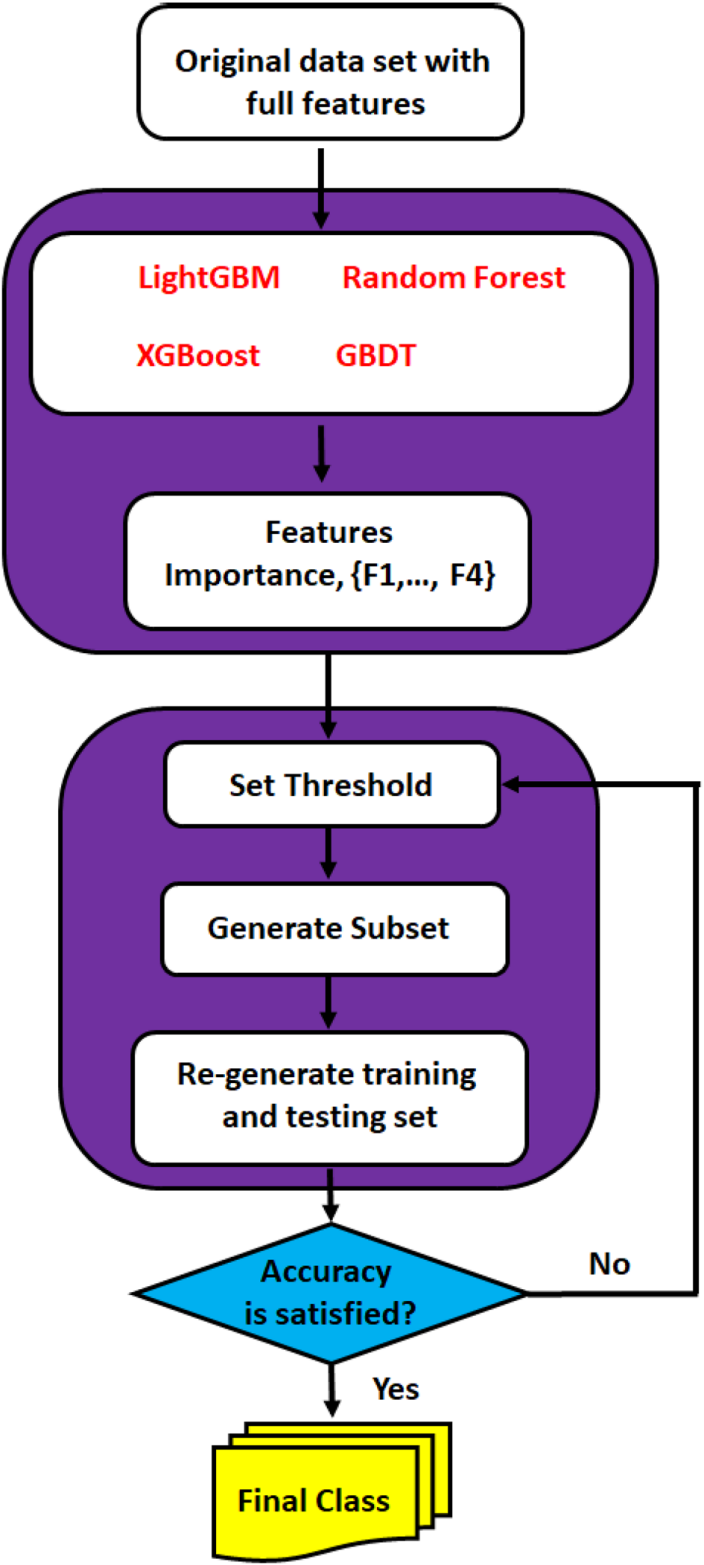
Feature selection and classification using different algorithm for predicting adsorption efficiency of arsenic by MOFs.

The affecting parameters on adsorptive performance are the initial concentration of arsenic (mg/L), adsorbent dosage (g/L), contact time (min), solution pH, temperature (°C), the specific surface area of MOFs (m^2^/g), and the presence of the anions. The statistical details of the dataset are listed in detail in Table 1. These parameters were considered as input data for the implemented models. At the same time, the removal percentage of As(V) was selected as the output of the models. Python, an open-source software, was used for modeling procedures. The training process of the models was performed using 85% of the data set called train subset. The performance of the models was investigated by 15% of the remaining dataset denoted test subset. Feature selection and classification using different algorithms for predicting the adsorption efficiency of As(V) by MOFs is presented in Fig. 2.

**Table 1:** Statistical details of the inputs and output parameters collected in this work.

	Surface area (m^2^/g)	Adsorbent dosage (g/L)	Arsenic concentration (mg/L)	Contact time (h)	Temperature (°C)	pH	Presence of anions	Removal efficiency (%)
Min	113.4	0.02	0.002	0.25	25	1.7	1	0.5
Max	1388	5	472.5	24	45	13	11	100

### Detection of outliers

Outliers are usually existed, especially in large datasets. The accuracy and reliability of the models can be significantly influenced by the outliers. Therefore, all datasets should be refined. In this work, the Leverage method was employed for detecting and eliminating outliers. The Hat matrix of the Leverage method was calculated based on Eq. (1)^58,59^:

1 $$H = Y\left( {Y^{T} Y} \right)^{ - 1} Y^{T}$$

In Eq. (1), Y is a matrix with $m \times n$ dimensions, where m is the number of experimental data and n stands for the number of input variables. The Hat matrix diagonal elements are the Hat value of data. Outliers can be recognized by developing William’s plot in which the normalized residuals are plotted versus the Hat values. The warning Leverage parameter $\left( {H^{*} } \right)$ is calculated based on Eq. (2) and is also shown in William’s plot^60^:

2 $$H^{*} = \frac{{3\left( {n + 1} \right)}}{m}$$

### Evaluation of the models quality

The quality and reliability of the developed models were assessed using different statistical techniques described as follows:

1. The average absolute relative deviation of the model results from the experimental values was calculated by the average absolute percent relative error (AAPRE) (Eq. (3)):
  3 $$AAPRE\left( \% \right) = \frac{100}{n}\mathop \sum \limits_{i = 1}^{n} \left| {\frac{{X\left( i \right)_{model} - X\left( i \right)_{exp} }}{{X\left( i \right)_{exp} }}} \right|$$
2. The root mean square error (RMSE), which indicates the error dispersion, is calculated by Eq. (4):
  4 $$RMSE = \left( {\frac{{\mathop \sum \nolimits_{i = 1}^{n} (X\left( i \right)_{model} - X\left( i \right)_{exp} )^{2} }}{n}} \right)^{{{\raise0.7ex\hbox{$1$} \!\mathord{\left/ {\vphantom {1 2}}\right.\kern-\nulldelimiterspace} \!\lower0.7ex\hbox{$2$}}}}$$
3. The dispersion of data is investigated by the standard deviation of errors (STD), which can be calculated using Eq. (5):
  5 $$STD = \frac{1}{n}\mathop \sum \limits_{i = 1}^{n} \left( {X\left( i \right)_{model} - \overline{X\left( i \right)}_{model} } \right)^{2} )^{{{\raise0.7ex\hbox{$1$} \!\mathord{\left/ {\vphantom {1 2}}\right.\kern-\nulldelimiterspace} \!\lower0.7ex\hbox{$2$}}}}$$
4. The coefficient of determination $\left( {R^{2} } \right)$ which assigns the accuracy of the predictions, can be calculated by Eq. (6). The $R^{2}$ value close to 1 determines that the estimation of experimental data is more accurate.
  6 $$R^{2} = 1 - \frac{{\mathop \sum \nolimits_{i = 1}^{n} \left( {X\left( i \right)_{model} - X\left( i \right)_{exp} } \right)^{2} }}{{\mathop \sum \nolimits_{i = 1}^{n} \left( {X\left( i \right)_{model} - \overline{X\left( i \right)}_{exp} } \right)^{2} }}$$

## Results and discussion

### Validation of the developed models

**Figure 3:**
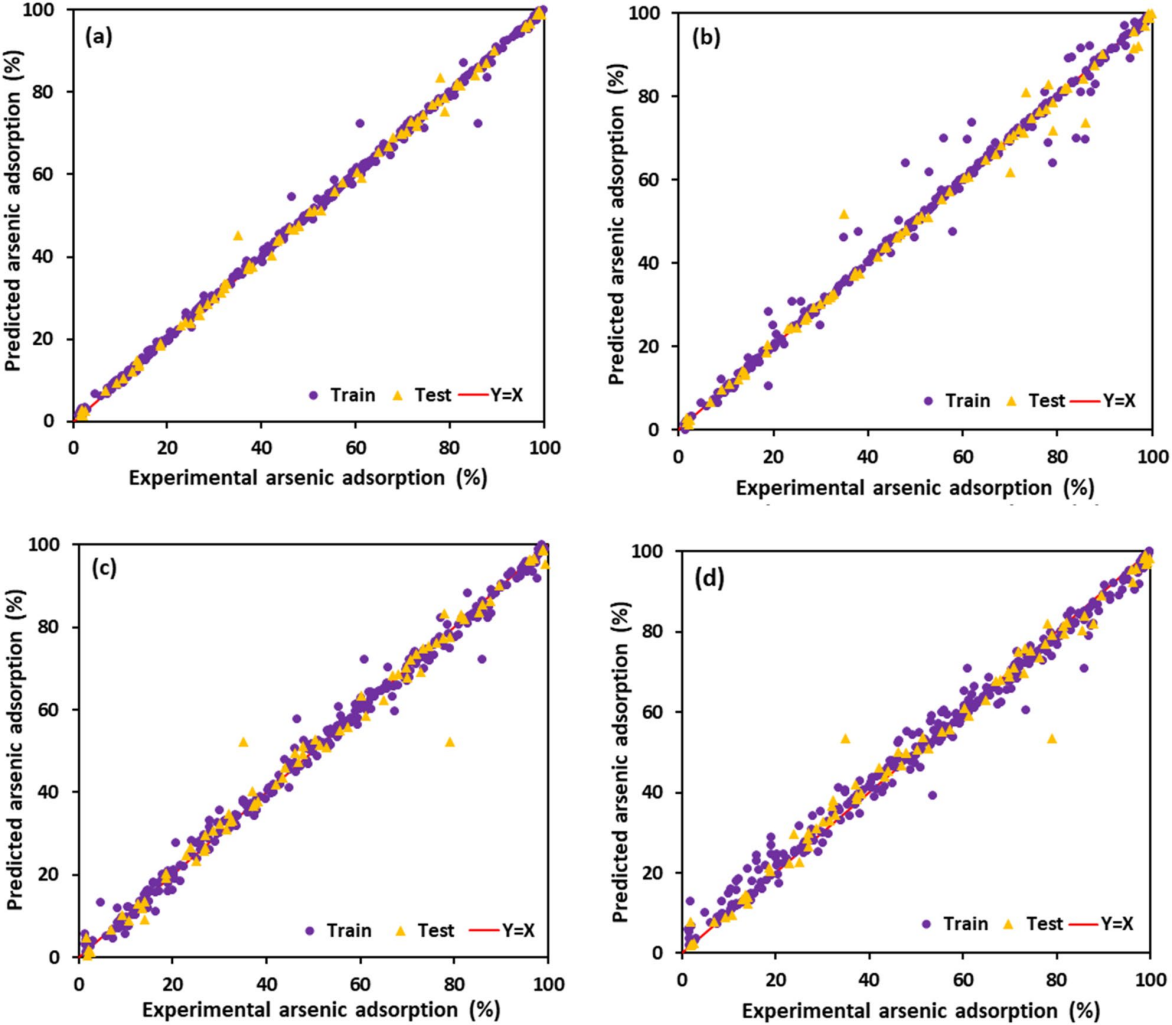
Cross plots of the proposed machine learning models in this study: (**a**) LightGBM, (**b**) XGBoost, (**c**) GBDT, and (**d**) RF.

**Figure 4:**
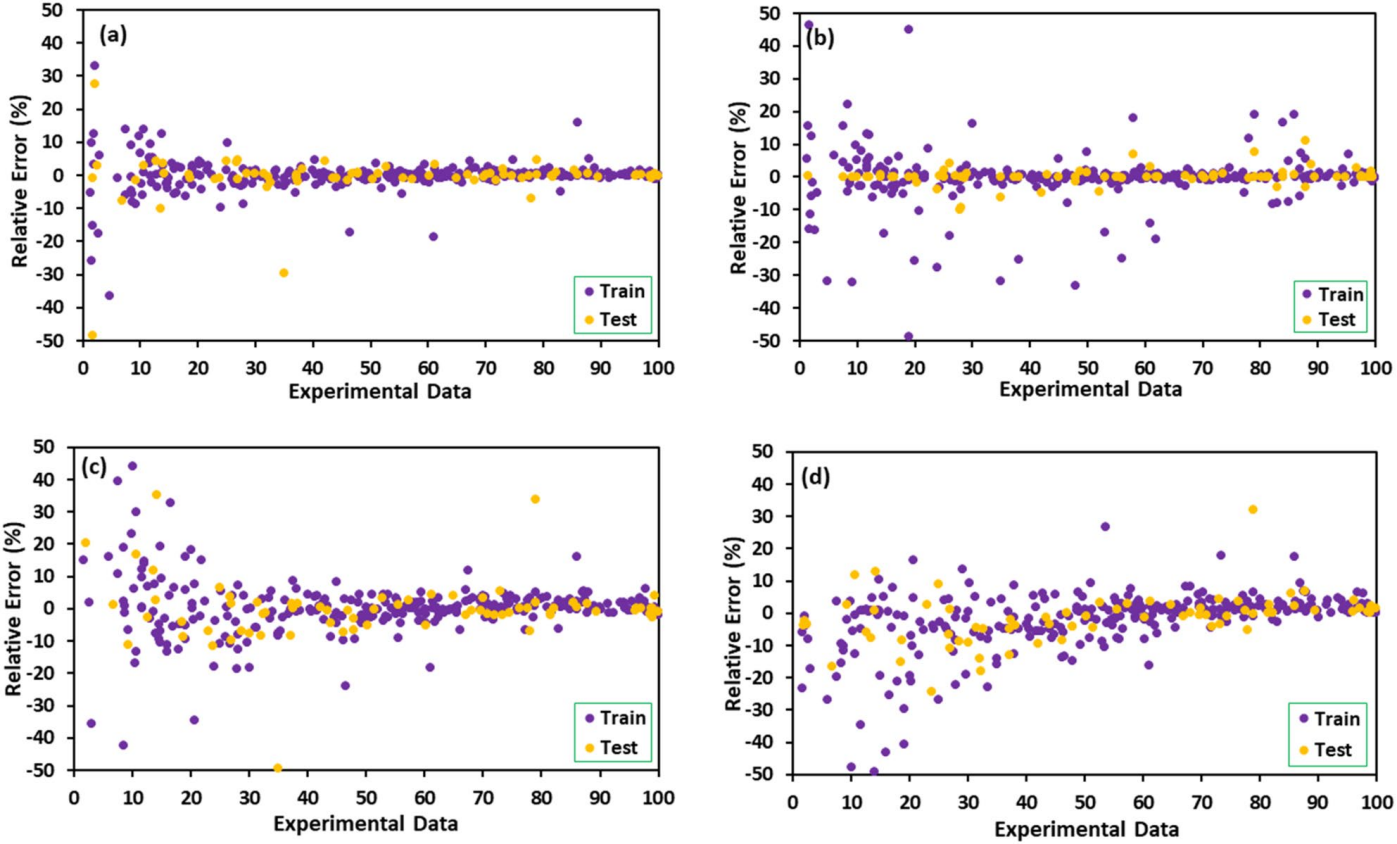
Error distribution plots of machine learning models for training and test sets: (**a**) LightGBM, (**b**) XGBoost, (**c**) GBDT, and (**d**) RF.

**Figure 5:**
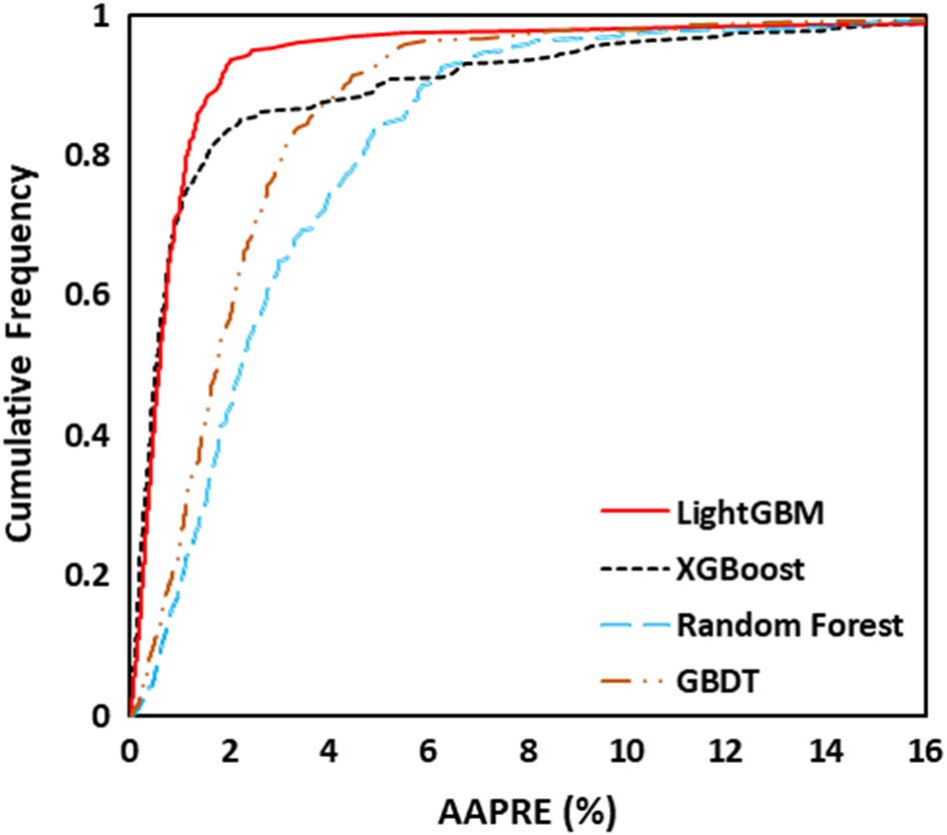
The cumulative frequency diagram of the proposed machine learning models.

The values of As(V) removal predicted by four developed models are illustrated in Fig. 3 regarding experimental data. When the predicted results are closer to the experimental data, the model with remarkable accuracy and reliability is achieved^61^. As shown in Fig. 3, all developed models revealed great accuracy where the data points scattered close enough to the line with a unit slope. It can be observed that the LightGBM model has excellently matched with experimental data amongst all the proposed models. The error distributions of all four developed models are depicted in Fig. 4. As shown, the errors fluctuated over zero line indicating that the models have been well developed with acceptable accuracy. However, the deviation of the LightGBM model from zero line was rarely notable compared with the others. The cumulative frequency of data versus AAPRE% is plotted in Fig. 5, visually indicating the model with higher accuracy. The model that is closest to the vertical axis is the most accurate. As can be seen, about 95% of data points can be predicted with AAPRE lower than 2% using the LightGBM and XGBoost. While, smaller than 40% of data points were predicted with AAPRE less than 2%, when RF and GBDT approaches were employed. Therefore, GBDT and RF models provided weak performance with less accuracy among the implemented models.

**Figure 6:**
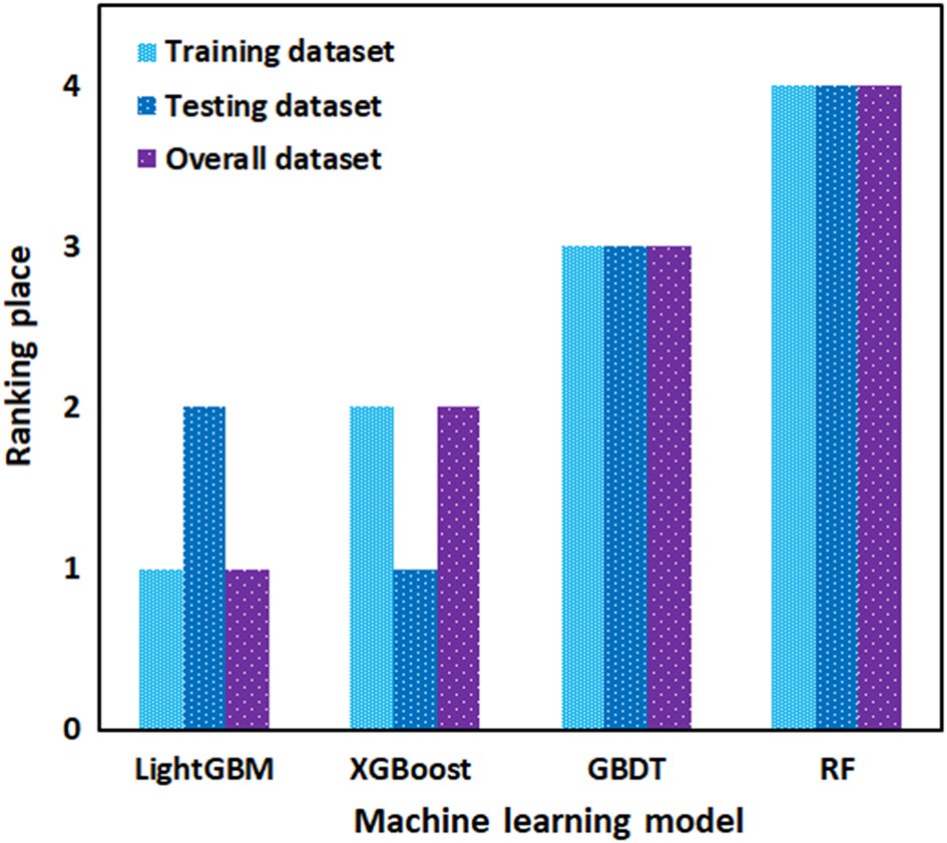
Comparison between AARPE of the developed models.

The performance of all developed models is also evaluated using additional statistical methods. Different statistical data attributed to the train, test and total data set of each model are listed in Table 2. With the highest total ${R}^{2}$ value of 0.9958 and the least RMSE value of 2.0688, the LightGBM model exhibited supreme performance. Also, based on the AAPRE% and STD, the LightGBM model presented the best performance. In the LightGBM model, the values of AAPRE and STD for train, test and total dataset were 2.11%, 0.0554, and 2.43%, and 0.0825, 2.88%, and 0.0628, respectively, which were the least obtained values among the developed models. In addition, the implemented models were selected before, and their accuracy in the training and testing stages and over the whole of the database was monitored using four statistical matrices. It is hard to most accurate one through visual inspection. Therefore, the ranking analysis is employed for doing so^62^. Figure 6 provides the results of model ranking in each stage based on the average values of the four statistical criteria reported in Table 2. The LightGBM model in the learning step is the best; nevertheless, it shows the second-ranking in the testing stage, and XGBoost depicts the best performance. On the other hand, the LightGBM with the first ranks over the whole database is the best model for predicting arsenic removal using MOFs. According to the results, the developed predictive models can be summarily ranked in terms of their accuracy as follows: LightGBM > XGBoost > GBDT > RF.

**Table 2:** Calculated statistical criteria for the developed models.

Statistical criteria		R^2^	RMSE	STD	AAPRE (%)
LightGBM	Train	0.9983	1.4013	0.0554	2.11
	Test	0.9852	3.6872	0.0825	2.43
	Total	0.9958	2.0688	0.0628	2.88
XGBoost	Train	0.9931	2.6873	0.0668	2.66
	Test	0.9832	3.7892	0.1007	3.52
	Total	0.9879	2.8081	0.0854	3.19
GBDT	Train	0.9922	2.3791	0.2731	8.55
	Test	0.9826	4.1125	0.2452	9.09
	Total	0.9812	2.9137	0.2698	8.72
RF	Train	0.9804	2.0364	0.4561	9.59
	Test	0.9762	4.4207	0.4233	10.72
	Total	0.9799	3.3845	0.4507	10.13

### Trend analysis of the LightGBM

**Figure 7:**
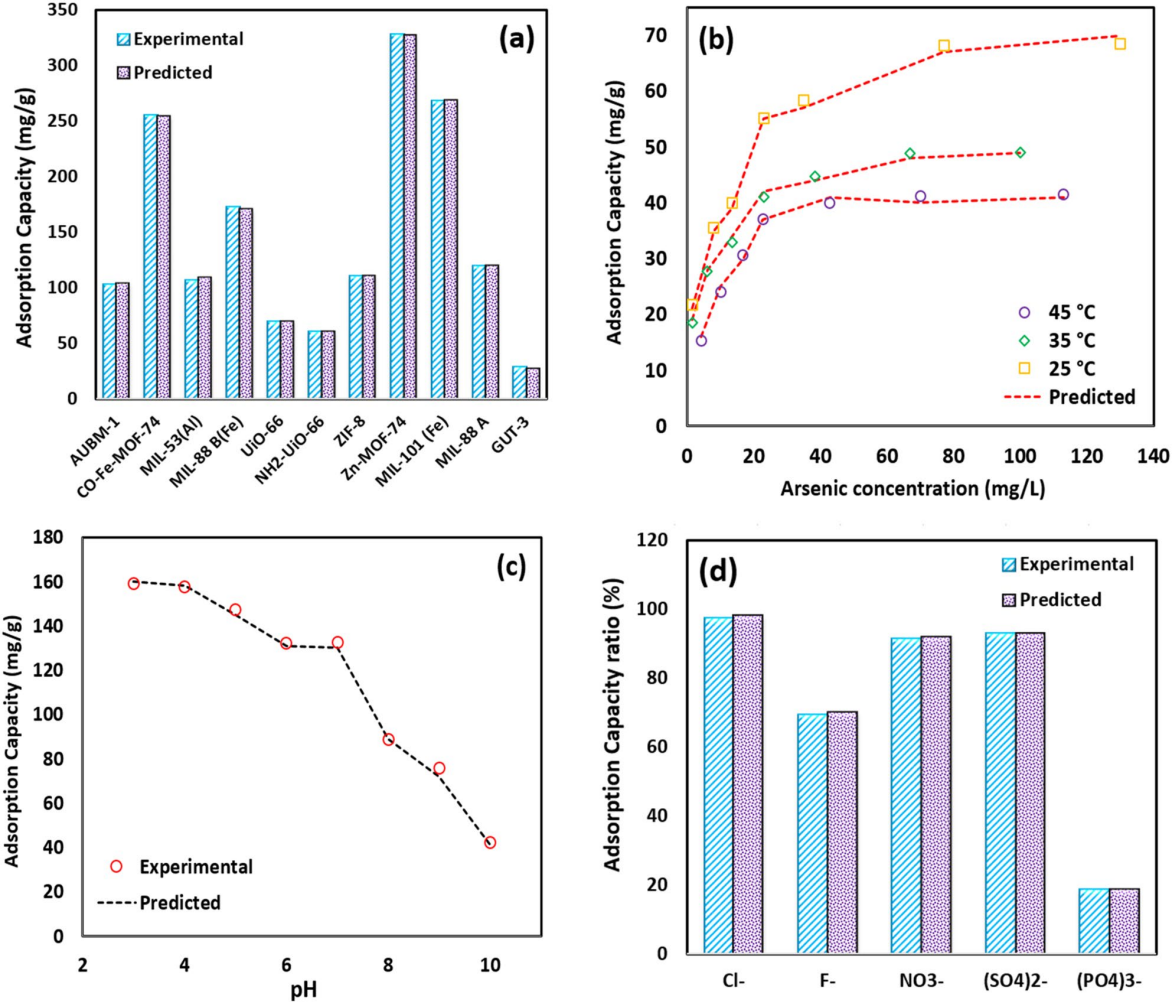
(**a**) The performance of different MOFs in the adsorption of As(V), (**b**) The effect of temperature on the adsorption capacity of UiO-66-NH_2_ in different As(V) concentration (pH = 9.2), (**c**) The effect of solution pH on arsenic removal by Fe-Co-MOF-74 (Initial arsenic concentration = 100 mg/L, adsorbent dose = 0.5 g/L, temperature = 25 °C), and (**d**) The effect of competition anions on the adsorption capacity of ZIF-8 (pH = 7).

The adsorptive removal of As(V) is highly influenced by operating conditions. In this section, the capability of the most accurate model, the LightGBM, was examined for predicting the trends of different parameters on the arsenic adsorption using various MOFs. In Fig. 7, the obtained results of the developed LightGBM model are compared with experimental data. The affecting parameters examined under different conditions were temperature, As(V) concentration, solution pH, and the coexistence of anions. As seen in Fig. 7, for all operating conditions, the LightGBM model exhibited excellent performance, predicting experimental data with high accuracy. Figure 7a illustrates the maximum adsorption capacity of As(V) obtained from different MOFs in comparison with predicted values by the LightGBM model. MOFs with different structural and morphological properties depicted various adsorption capacities, but interestingly, the performance of MOFs could be predicted with excellent accuracy using the developed model. Comparison between all mentioned MOFs, the Zn-MOF-74 had a maximum capacity of 328 mg/g because of its high surface area (604 m^2^/g), nearly twelve times of GUT-3 (209 m^2^/g) with the adsorption capacity of 29 mg/g. It has also been found that the amount of arsenic adsorption on the MOF structure is directly attributed to surface properties (e.g., charge, functional group, morphology, etc.) besides the surface area.

Another important factor influencing the adsorption capacity of adsorbents is the initial concentration of pollutants. As shown in Fig. 7b, the effects of As(V) initial concentration on the adsorption capacity of UiO-66-NH_2_ at different temperatures^51^ can be precisely predicted using the developed LightGBM approach. It can be seen that the adsorption capacity of the adsorbent increased with increasing the initial concentration of As(V) due to the more available metal ions present in the solution. Moreover, the enhancement of temperature from 25 to 45 °C resulted in a steady decrease in the adsorptive removal efficiency of As(V) over UiO-66-NH_2_. This observation confirms that As(V) adsorption is exothermic, therefore increasing temperature probably weakens the binding forces formed between the pollutants and the active sites of the adsorbent^63^.

The solution pH is a very critical parameter affecting the adsorptive removal of different pollutants. The pH can influence the surface potential of the adsorbent as well as the type of the pollutant species in the solution^64^. The effect of different solution pH on the As(V) removal over Fe-Co-based MOF-74^4^ are compared with the estimated results by the LightGBM model in Fig. 7c. As depicted, the adsorption capacity indicated a steady decreasing trend with increasing pH from 3 to 10. Based on the obtained results, in the examined pH range, the adsorbent maintained a positive surface charge, while, As(V) existed in the form of negatively charged species^4^. Thus, the electrostatic interaction between As(V) and Fe-Co based MOF-74 can explain the adsorption process. The decline in the adsorption capacity with increasing the solution pH can be assigned to the decreasing adsorbent surface potential^4^. As seen in Fig. 7c, the LightGBM model was a reliable technique providing accurate predictions for adsorption capacity in the whole examined pH.

The effect of the coexistence of various anions such as $NO_{3}^{ - } ,PO_{4}^{3 - } ,Cl^{ - } ,SO_{4}^{2 - } ,F^{ - }$ on the adsorption capacity of As(V) using ZIF-8^65^ was compared with the predicted results of the LightGBM model in Fig. 7d. It can be seen that $PO_{4}^{3 - }$ exhibited an intense inhibitory impact on the adsorption process. This may be due to the similar structure of $PO_{4}^{3 - }$ with $AsO_{4}^{3 - }$ and their competition for adsorption over active sites of MOFs. The presence of $F^{ - }$ also revealed the adverse effects on the As(V) removal. While the negative effects of other ions were negligible. As it is obvious in Fig. 7d, the implemented LightGBM approach was strongly capable for predicting the impact of coexistence of anions on the adsorption of As(V).

### The applicability domain of the LightGBM model

**Figure 8:**
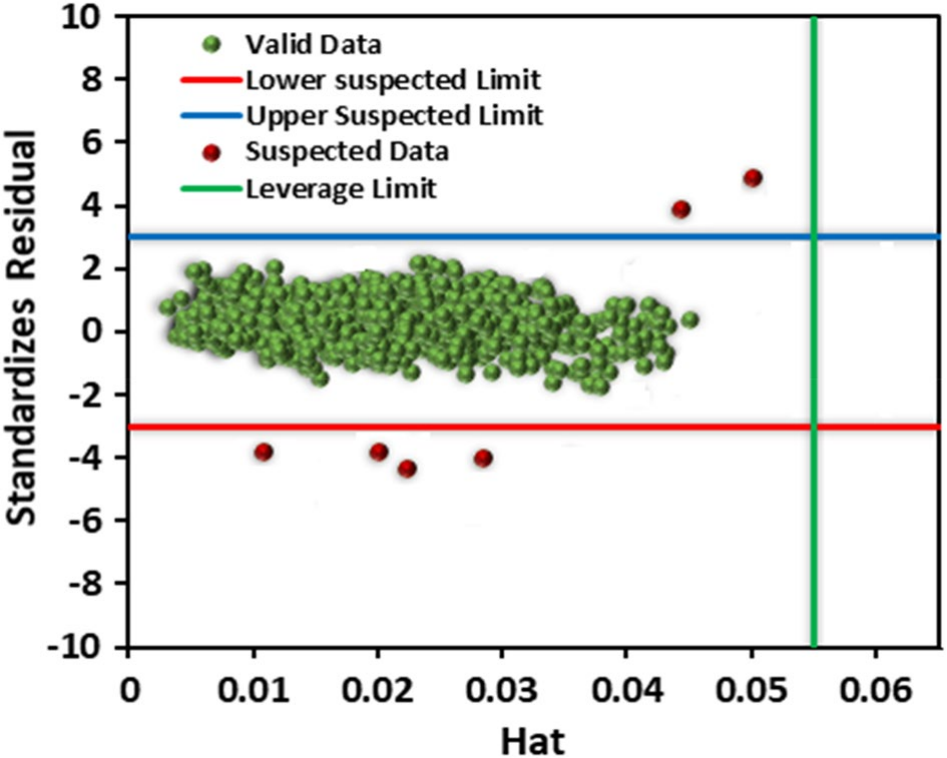
Outlier detection using William’s plot for the LightGBM model.

To evaluate the applicability area of the LightGBM model with the best performance, William’s plot was plotted in Fig. 8. In the Leverage approach, the data in the area determined by standard residuals of + 3 to − 3 on the y-axis and 0 to H* on the x-axis are only valid. Based on Fig. 8, only six data points were placed out of the valid domain of William’s plot. Accordingly, these points were considered experimentally uncertain data. Since the number of doubtful data was small compared to the entire data set, it can be concluded that both collected experimental data and the LightGBM model were statistically valid and trustworthy.

### The sensitivity analysis

The sensitivity analysis was performed to evaluate the magnitude of the impacts of all input parameters on the As(V) removal predicted by the LightGBM model^66^. The value of the relevancy factor (r) determines the extent of each input parameter’s effect on the As(V) adsorption^67^. The factor “r” can be a negative or positive value. A positive value for unique input data confirms that the output variable directly interacts with that input data. Whereas the negative value reveals the inverse interaction between output and input variable^68^. In addition, the greater the absolute value of “r” for a particular input parameter, the more significant the impact of that variable on the model output^69^. The relevancy factor is computed by Eq. (7):

7 $$r\left( {I_{i} ,\omega } \right) = \frac{{\mathop \sum \nolimits_{j = 1}^{N} \left( {I_{ij} - \overline{{I_{i} }} } \right)\left( {\omega_{j} - \overline{\omega }} \right)}}{{\left( {\mathop \sum \nolimits_{j = 1}^{N} \left( {I_{ij} - \overline{{I_{j} }} } \right)^{2} \mathop \sum \nolimits_{j = 1}^{n} \left( {\omega_{j} - \overline{\omega }} \right)^{2} } \right)^{0.5} }}$$

where $\omega_{j}$ and $\overline{\omega }$ are the jth and the mean value of the predicted As(V) removal, respectively. $I_{ij}$ and $\overline{{I_{i} }}$ represent the ith and the mean value of the ith input variable, respectively. N is the total number of data.

**Figure 9:**
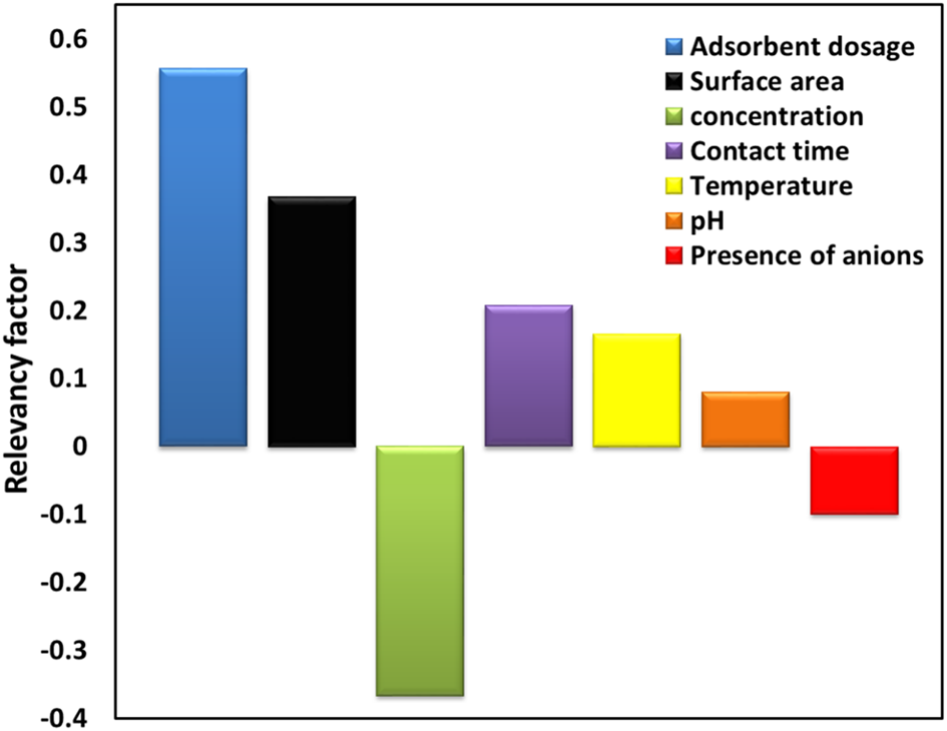
Sensitivity analysis for the developed LightGBM model on different input variables.

The calculated relevancy factor of all input parameters on the As(V) adsorption predicted by the LightGBM model is plotted in Fig. 9. As mentioned above, the input parameters were MOFs surface area, adsorbent dosage, arsenic concentration, contact time, temperature, solution pH, and presence of anions. As illustrated in Fig. 9, the adsorbent dosage and surface area of the MOFs exhibited the most positive impacts on As(V) adsorption. This confirms that any increase in the adsorbent content, as well as the adsorbent specific surface area would result in increasing the amount of As(V) removal. On the other hand, the initial arsenic concentration inversely influenced the adsorption process. Other parameters such as the presence of anions and pH had negligible effects on the model output.

## Conclusion

In this study, the potential of four different ML approaches, LightGBM, XGBoost, GBDT, and RF, were investigated to estimate As(V) adsorption from wastewater. An experimental dataset of As(V) removal using 13 different MOFs was selected with various operating conditions. Validation of the proposed models was performed using statistical methods. The LightGBM model with the least AAPRE value of 2.88% and the least STD value of 0.0628 was the most trustworthy model. Based on the cumulative frequency diagram of the LightGBM model, about 95% of data points can be estimated with AAPRE lower than 2%. In addition, the Leverage approach proved that most of the data points of the LightGBM model were scattered within the valid domain of William’s plot. Moreover, the effects of different operating parameters such as initial arsenic concentration, temperature, solution pH, and the presence of anions can be predicted accurately on the As(V) removal. This study confirms ML approaches that are cost affordable and straightforward can be effectively employed for wastewater treatment.

## Supplementary Information

Supplementary Information.

## Data Availability

The collected experimental databank has been added to the manuscript (please see Supplementary Information: Dataset).
